# COVID-19 Vaccination Among US-Born and Non–US-Born Residents of the United States From a Nationally Distributed Survey: Cross-sectional Study

**DOI:** 10.2196/43672

**Published:** 2023-06-20

**Authors:** Francisco Alejandro Montiel Ishino, Kevin Villalobos, Faustine Williams

**Affiliations:** 1 Division of Intramural Research Epidemiology Branch National Institute of Environmental Health Sciences Durham, NC United States; 2 Division of Intramural Research National Institute on Minority Health and Health Disparities Bethesda, MD United States

**Keywords:** COVID-19, vaccination, nativity, health disparity, survey, sociodemographics, socioeconomics, pandemic, vaccine, US, USA, United States, birth, country

## Abstract

**Background:**

Extended literature has demonstrated that COVID-19 vaccination is crucial for the health of all individuals, regardless of age. Research on vaccination status in the United States (US) among US-born and non–US-born residents is limited.

**Objective:**

The objective of our study was to examine COVID-19 vaccination during the pandemic among US-born and non–US-born people, while accounting for sociodemographic and socioeconomic factors gathered through a nationally distributed survey.

**Methods:**

A descriptive analysis was conducted on a comprehensive 116-item survey distributed between May 2021 and January 2022 across the US by self-reported COVID-19 vaccination and US/non-US birth status. For participants that responded that they were not vaccinated, we asked if they were “not at all likely,” “slightly to moderately likely,” or “very to extremely likely” to be vaccinated. Race and ethnicity were categorized as White, Black or African American, Asian, American Indian or Alaskan Native, Hawaiian or Pacific Islander, African, Middle Eastern, and multiracial or multiethnic. Additional sociodemographic and socioeconomic variables included gender, sexual orientation, age group, annual household income, educational attainment, and employment status.

**Results:**

The majority of the sample, regardless of whether they were US-born or non–US-born, reported being vaccinated (3639/5404, 67.34%). The US-born participants with the highest proportion of COVID-19 vaccination self-identified as White (1431/2753, 51.98%), while the highest proportion of vaccination among non–US-born participants was found among participants who self-identified as Hispanic/Latino (310/886, 34.99%). Comparing US-born and non–US-born participants showed that among those who were not vaccinated, the highest self-reported sociodemographic characteristics by proportion were similar between the groups, and included identifying as a woman, being straight or heterosexual, being aged 18 to 35 years, having an annual household income <$25,000, and being unemployed or taking part in nontraditional work. Among the 32.66% (1765/5404) of participants that reported not being vaccinated, 45.16% (797/1765) stated that they were not at all likely to seek vaccination. Examining US/non–US birth status and the likelihood to be vaccinated for COVID-19 among nonvaccinated participants revealed that the highest proportions of both US-born and non–US-born participants reported being not at all likely to seek vaccination. Non–US-born participants, however, were almost proportionally distributed in their likelihood to seek vaccination; they reported to be “very to extremely likely” to vaccinate (112/356, 31.46%); compared to 19.45% (274/1409) of US-born individuals reporting the same.

**Conclusions:**

Our study highlights the need to further explore factors that can increase the likelihood of seeking vaccination among underrepresented and hard-to-reach populations, with a particular focus on tailoring interventions for US-born individuals. For instance, non–US-born individuals were most likely to vaccinate when reporting COVID-19 nonvaccination than US-born individuals. These findings will aid in identifying points of intervention for vaccine hesitancy and promoting vaccine adoption during current and future pandemics.

## Introduction

Currently, 68% of the US population is vaccinated for COVID-19, with approximately 609 million doses having been given [1]. While intentions, attitudes, and barriers play a role in vaccination in the United States (US), the contribution of race/ethnicity and US/non–US birth status to vaccine uptake is not well understood in the context of COVID-19. Multiple factors, such as affiliations and folk beliefs that affect conventional vaccination schedules, may affect intentions toward COVID-19 vaccination [2]. The role of US/non–US birth status in the US and COVID-19 vaccination, however, is relatively unknown, although some research indicates that US/non–US birth status and race/ethnicity could be crucial when evaluating vaccination intentions. For example, Burger et al [3] explored the influence of Hispanic ethnicity and US/non–US birth status on H1N1 pandemic vaccination uptake and discovered that both US-born and non–US-born Hispanic people were less likely to receive H1N1 vaccination when compared to non-Hispanic White people. While US-born Hispanic vaccination hesitance is mostly explained by sociodemographic reasons, such as age and marital status, hesitancy among non–US-born Hispanic people is attributed to socioeconomics and resource availability. By contrast, Frisco et al [4] found that US-born Black adults were more hesitant about COVID-19 vaccination compared to US-born White adults, while US-born Hispanic adults were less vaccine hesitant than US-born White adults.

Furthermore, it is well documented that minority groups such as Hispanic/Latino and Black people are disproportionately affected by chronic or comorbid diseases. We also understand that individuals with certain medical conditions, such as heart disease or diabetes mellitus, are at higher risk of developing dangerous COVID-19 symptoms and other related infections [5]. According to the US Centers for Disease Control and Prevention, American Indian/Alaska Native, Black/African American, and Hispanic/Latino people in the US are at increased risk of COVID-19 infection, hospitalization, and death due to chronic comorbid conditions compared to White people [6]. Hence, vaccination uptake could be a critical determinant of health. Exploring vaccination patterns in the US is an important public health concern. Therefore, we used a nationally distributed survey to capture a cross-sectional sample of the US to explore patterns in COVID-19 vaccination. Our purpose was to expand on the sparse literature regarding the COVID-19 pandemic and vaccination among US-born and non–US-born individuals while accounting for diverse socioeconomic and sociodemographic factors.

## Methods

### Overview

A survey was distributed nationally as part of a study, Understanding the Impact of the Novel Coronavirus (COVID-19) and Social Distancing on Physical and Psychosocial (Mental) Health and Chronic Diseases. US residents aged 18 years and older, both US-born and non–US-born, were our target population. Inclusion of racial and ethnic groups was our main priority, including Hispanic/Latino, White, Black, Asian, American Indian/Alaskan Native, and Native Hawaiian/Pacific Islander participants. Low income (ie, less than US $25,000 in annual household income) and being a rural resident were oversampled among non-Hispanic White, non-Hispanic Black, Hispanic, and non–US-born participants. Qualtrics (Qualtrics LLC) was contracted to facilitate the recruitment and distribution of the web-based survey across the US. Qualtrics distributed 10,000 surveys between May 13, 2021, and January 9, 2022. After expert review for fraud detection by Qualtrics, 5938 surveys were received by the research team. Montiel Ishino et al [7] describe further information on the survey design, participant recruitment, and implementation. A total of 5404 participants who completed the 116-item survey were included in this study, which cut across multiple domains, including, but not limited to, COVID-19 symptoms, testing and prevention, pandemic economic impact, and sociodemographics.

### Ethical Considerations

The Understanding the Impact of the Novel Coronavirus (COVID-19) and Social Distancing on Physical and Psychosocial (Mental) Health and Chronic Diseases study was directed by author FW. Web-based informed consent was obtained from participants before they took part in the survey as part of the recruitment process by Qualtrics. If participants decided to take part, we reassured them that their responses would be kept confidential and that their participation was completely voluntary. As such, participants could choose to stop taking part in the study at any time or not answer any questions they did not want to without fear of repercussion. Participants received a US $10 gift card after the completion of the survey as compensation for 30 minutes of their time. Participants were provided the contact information of the principal investigator (FW) and the phone number of the National Institutes of Health (NIH) Institutional Review Board.

The research protocol for this study was reviewed by the NIH Intramural Research Program Institutional Review Board, Human Research Protection Program, and Office of Human Subjects Research Protections and received an exemption on December 23, 2020 (000308). The NIH, Intramural Research Program Institutional Review Board, Human Research Protection Program, and Office of Human Subjects Research Protections determined that our protocol did not involve human participants and was excluded from institutional review board review.

## Results

The majority of the sample of US-born participants that reported being vaccinated self-identified as White (1431/2753, 51.98%); were women (1667/2752, 60.57%); were straight or heterosexual (2474/2739, 90.32%); were aged 36 to 55 years (1043/2648, 39.39%); had an annual household income of US $75,000 or more (840/2723, 30.85%); had some college education, an associate degree, vocational training, or technical training (907/2750, 32.98%); and were unemployed or taking part in nontraditional work (1178/2752, 42.81%). Most of the vaccinated non–US-born sample self-reported as Hispanic/Latino (310/886, 34.99%), were women (555/886, 62.64%), were straight or heterosexual (767/880, 87.16%), were aged between 18 to 35 or 36 to 55 years (both 300/883, 37.17%), had an annual household income of $75,000 or more (290/878, 33.03%), had a bachelor’s degree (283/883, 32.05%), and were unemployed or participating in nontraditional work (1178/1408, 44.91%).

The US-born sample that had not received the COVID-19 vaccine primarily self-identified as White (621/1409, 44.07%); were women (918/1405, 65.34%); were straight or heterosexual (1251/1401, 89.29%); were aged 18 to 35 years (633/1340, 47.24%); had an annual household income of less than US $25,000 (445/1396, 31.88%); had some college education, an associate degree, or vocational or technical training (541/1408, 38.42%); and were unemployed or taking part in nontraditional work (640/1406, 45.52%). The non–US-born sample of those that had not received the COVID-19 vaccine self-identified as Hispanic/Latino (123/356, 34.55%), were women (221/354, 62.43%), were straight or heterosexual (305/353, 86.40%), were aged 18 to 35 years (176/315, 55.87%), had an annual household income of less than US $25,000 (116/350, 33.14%), had a high school diploma or had taken the General Educational Development (GED) test (94/354, 26.55%), and were unemployed or taking part in nontraditional work (180/354, 50.85%). Table 1 shows more detail.

**Table 1:** Characteristics of COVID-19 vaccination sample by country of birth (n=5404).

			Vaccinated (n=3639), n (%)					Not vaccinated (n=1765), n (%)				*χ*^2^ (*df*)	
			US-born		Non–US-born		US-born		Non–US-born				
**Race/ethnicity**													—^a^
	White	1431 (51.98)		91 (10.27)		621 (44.07)			37 (10.39)				
	Black/African American	592 (21.5)		107 (12.08)		431 (30.59)			74 (20.79)				
	Hispanic/Latino	361 (13.11)		310 (34.99)		188 (13.34)			123 (34.55)				
	Asian	177 (6.43)		279 (31.49)		40 (2.84)			57 (16.01)				
	American Indian/Alaska Native	76 (2.76)		5 (0.56)		61 (4.33)			3 (0.84)				
	Hawaiian/Pacific Islander	35 (1.27)		7 (0.79)		12 (0.85)			7 (1.97)				
	African	18 (0.65)		14 (1.58)		10 (0.71)			12 (3.37)				
	Middle Eastern	4 (0.15)		12 (1.35)		3 (0.21)			9 (2.53)				
	Multiracial/multiethnic	59 (2.14)		61 (6.88)		43 (3.05)			34 (9.55)				
**Gender^b^**													49.85 (6)
	Man	1029 (37.39)		303 (34.2)		464 (33.02)			108 (30.51)				
	Woman	1667 (60.57)		555 (62.64)		918 (65.34)			221 (62.43)				
	Nonbinary, transgender, or other	56 (2.03)		28 (3.16)		23 (1.64)			25 (7.06)				
**Sexual orientation^b^**													31.75 (9)
	Straight/heterosexual	2474 (90.32)		767 (87.16)		1251 (89.29)			305 (86.4)				
	Lesbian or gay	101 (3.69)		39 (4.43)		46 (3.28)			10 (2.83)				
	Bisexual	139 (5.07)		59 (6.7)		87 (6.21)			24 (6.8)				
	Other	25 (0.91)		15 (1.7)		17 (1.21)			14 (3.97)				
**Age group (years)^b^**													244.79 (6)
	18 to 35	784 (29.61)		300 (37.17)		633 (47.24)			176 (55.87)				
	36 to 55	1043 (39.39)		300 (37.17)		532 (39.7)			90 (28.57)				
	56 to 85 and older	821 (31)		207 (25.65)		175 (13.06)			49 (15.56)				
**Household income (US $)^b^**													217.29 (12)
	<$25,000	583 (21.41)		158 (18)		445 (31.88)			116 (33.14)				
	$25,000 to $34,999	382 (14.03)		133 (12.87)		263 (18.84)			31 (17.43)				
	$35,000 to $49,999	384 (14.1)		138 (15.72)		253 (18.12)			54 (15.43)				
	$50,000 to $74,999	534 (19.61)		179 (20.39)		220 (15.76)			60 (17.14)				
	$75,000 or more	840 (30.85)		290 (33.03)		215 (15.4)			59 (16.86)				
**Educational attainment^b^**													412.26 (12)
	Less than high school	104 (3.78)		46 (5.21)		117 (8.31)			53 (14.97)				
	High school or General Education Development test	559 (20.33)		143 (16.19)		448 (31.82)			94 (26.55)				
	Some college, vocational/technical training	907 (32.98)		211 (23.9)		541 (38.42)			83 (23.45)				
	Bachelor’s degree	812 (29.53)		283 (32.05)		219 (15.55)			88 (24.86)				
	Master’s degree or above	368 (13.38)		200 (22.65)		83 (5.89)			36 (10.17)				
**Employment^c^**													16.98 (6)
	Unemployed or nontraditional work	1178 (42.81)		397 (44.91)		640 (45.52)			180 (50.85)				
	Nonessential worker	853 (31)		284 (32.13)		446 (31.72)			109 (30.79)				
	Essential worker	721 (26.19)		203 (22.96)		320 (22.76)			65 (18.36)				

^a^Not available; neither the chi-square test nor the Fisher exact test could be computed.

^b^*P*<.001.

^c^*P*<.01.

We found that there were statistically significant relationships between vaccination status and being US-born (*χ*^2^_1_=11.72, n=5404; *P*<.01) and, among participants who were not vaccinated, between vaccination likelihood and being US-born (*χ*^2^_2_=26.68, n=1765; *P*<.001). As seen in Table 2, 32.66% (1765/5404) of the total sample reported not having been vaccinated for COVID-19. Of those unvaccinated, the majority reported that they were “not likely at all” to seek vaccination (797/1765, 45.16%). When split up by country of birth, the highest proportions of both US-born and non–US-born participants reported being “not likely at all” to seek vaccination. The proportion of non–US-born participants that reported they were “very to extremely likely” to seek vaccination was 31.46% (112/356), which was higher than among US-born participants (274/1409, 19.45%).

**Table 2:** COVID-19 vaccination status and likelihood of vaccination by country of birth.

		US-born, n (%)		Non–US-born, n (%)		Total, n (%)
**Vaccinated (n=5404)^a^**						
	Yes	2753 (66.15)	886 (71.34)		3639 (67.34)	
	No	1409 (33.85)	356 (28.66)		1765 (32.66)	
**If not vaccinated, likelihood of seeking vaccination (n=1765)^b^**						
	Not at all likely	668 (47.41)	129 (36.24)		797 (45.16)	
	Slightly to moderately likely	467 (33.14)	115 (32.30)		582 (32.97)	
	Very to extremely likely	274 (19.45)	112 (31.46)		386 (21.87)	

^a^*P*=.001 (chi-square test).

^b^*P*<.001 (chi-square test).

## Discussion

### Principal Findings

Proportionally, the majority of US-born and non–US-born individuals that reported COVID-19 vaccination were observed to be women, reported an income of $75,000 or more, and were unemployed or taking part in nontraditional work. The sample of non–US-born vaccinated individuals was primarily Hispanic/Latino, followed closely by Asian people, whereas White people were the highest proportion in the US-born group. One distinction between the groups was that among the non–US-born vaccinated group, those reporting having a bachelor’s degree had the highest proportion of being vaccinated, followed by those with a master’s or higher degree. This is in contrast to the US-born vaccinated group, which primarily had education at the college, associate degree, or vocational/technical level, followed by the bachelor’s degree level.

While the US-born sample showed similarities between those who were vaccinated and not vaccinated, the unvaccinated overall were young (ie, between the ages of 18 to 35 years) and reported an income of less than US $25,000. These findings regarding lower income status have also been seen in previous studies that explored vaccination and socioeconomic factors [8-10]. Both the vaccinated and unvaccinated US-born participants were also similar that in the largest proportion of reported educational attainment was some college education, an associate degree, or vocational or technical training; however, the groups differed in that the second-largest proportion of reported educational attainment was having a high school diploma or GED test. The largest proportion of US-born vaccinated participants were those with some college education, an associate degree, or vocational or technical training, whereas non–US-born vaccinated participants most often reported having a bachelor’s degree. The non–US-born unvaccinated sample had a greater proportion of participants with a bachelor’s, master’s, or higher degree than the US-born sample. The unvaccinated US-born sample was observed to have the second highest proportion of Black/African American participants. This agrees with prior research that indicates that Black/African American people are more likely to be hesitant to take the COVID-19 vaccine and, due to limited access, less likely to receive it [11]. Proportionally, at approximately 35%, non–US-born Hispanic/Latino people had similar proportions of COVID-19 vaccination and nonvaccination.

Overall, the highest proportion of participants who were nonbinary, transgender, or reported having a gender of “other” was observed among those who were unvaccinated and non–US-born. More research is needed to understand if this observation is due to liminal identities or possible disparities in access or informational needs. Future studies need to focus on oversampling medically underserved and underrepresented groups, particularly among non–US-born subpopulations. For instance, there may be salient interventions that can be tailored and developed for non–US groups. We observed that while unvaccinated US-born participants were more proportionally skewed to be “not at all likely” followed by “slightly to moderately likely” to seek a COVID-19 vaccine, non–US-born participants were almost equally distributed in their likelihood of seeking vaccination. Non–US-born participants, if provided the correct intervention, may elect to seek the COVID-19 vaccine and those in the “slightly to moderately likely” group may be swayed to the “extremely likely” group.

### Conclusion

We conducted the cross-sectional study Understanding the Impact of the Novel Coronavirus (COVID-19) and Social Distancing on Physical and Psychosocial (Mental) Health and Chronic Diseases using a nationally distributed survey to understand multiple factors behind COVID-19 vaccination. Our findings indicate that the majority of our participants were vaccinated and that those who were not vaccinated were highly unlikely to seek vaccination. The results also indicate that socioeconomic factors such as income affect vaccination uptake. The results from this study can be used as evidence and as an initial step for further analysis to promote health equity among vulnerable groups.

## Abbreviations

GED: General Education Development

## Data Availability

The survey data from the Understanding the Impact of the Novel Coronavirus (COVID-19) and Social Distancing on Physical and Psychosocial (Mental) Health and Chronic Diseases study are available upon request to author FW.
